# Cognitive Collaboration Found in Cardiac Physiology: Study in Classroom Environment

**DOI:** 10.1371/journal.pone.0159178

**Published:** 2016-07-14

**Authors:** Lauri Ahonen, Benjamin Cowley, Jari Torniainen, Antti Ukkonen, Arto Vihavainen, Kai Puolamäki

**Affiliations:** 1 Finnish Institute of Occupational Health, Helsinki, Finland; 2 Department of Computer Science, University of Helsinki, Helsinki, Finland; 3 Cognitive Brain Research Unit, Institute of Behavioural Sciences, University of Helsinki, Helsinki, Finland; IRCCS Istituto Auxologico Italiano, ITALY

## Abstract

It is known that periods of intense social interaction result in shared patterns in collaborators’ physiological signals. However, applied quantitative research on collaboration is hindered due to scarcity of objective metrics of teamwork effectiveness. Indeed, especially in the domain of productive, ecologically-valid activity such as programming, there is a lack of evidence for the most effective, affordable and reliable measures of collaboration quality. In this study we investigate synchrony in physiological signals between collaborating computer science students performing pair-programming exercises in a class room environment. We recorded electrocardiography over the course of a 60 minute programming session, using lightweight physiological sensors. We employ correlation of heart-rate variability features to study social psychophysiological compliance of the collaborating students. We found evident physiological compliance in collaborating dyads’ heart-rate variability signals. Furthermore, dyads’ self-reported workload was associated with the physiological compliance. Our results show viability of a novel approach to field measurement using lightweight devices in an uncontrolled environment, and suggest that self-reported collaboration quality can be assessed via physiological signals.

## Introduction

In modern knowledge work and in higher education many scenarios arise that require collaborative problem solving—for example, complex programming assignments. The field of computer-supported collaborative work (CSCW) addresses this need, and the increased popularity of collaboration software (e.g., online editing tools) has led to increased interest in synchronous collaboration benefits and challenges [1].

Ackerman [2] has argued that collaborative computer systems need to support the “flexible, nuanced” social interaction patterns which underlie collaboration. However there remains a lack of solutions to harness data from the patterns which arise between collaborating individuals. An abundance of studies suggest that periods of intense social interaction result in congruent physiological response patterns in collaborators’ physiological signals [3]. Thus we argue that physiological patterns provide valuable data for CSCW systems, where highly synchronized physiological signals may indicate collaborators whose interaction is more likely to be productive. However there is a lack of such objective physiological indicators for effectiveness of pair and team work in naturalistic settings.

Especially in the domain of a productive, ecologically-valid task such as pair-programming in the classroom, there is an opportunity to discover and apply the most effective, affordable and reliable of such measures. One example of such a measure is the extent of synchrony between collaborators’ same physiological signals, e.g., heart-rate variability (HRV). We contend that if the signal in question has a proven relationship to cognitive activity, then in the context of a shared cognitive task, the degree to which such signals are synchronized can be used as an efficient means to identify relevant parameters of collaboration. It has the added benefit of working unobtrusively in real-time.

The aim of this paper is to evaluate the feasibility and relevance of synchronized physiological signals between collaborating individuals during naturalistic pair-work. Such synchrony has been termed *physiological compliance* [4]. In this study, we investigated physiological compliance between dyads collaborating on a pair-programming task, in a classroom setting with novice computer science students. This is a completely naturalistic setting since social interaction is unconstrained, and the students worked on programming assignments taken from their standard curriculum. We measured the classroom on four different occasions using practical recording methods. Practicality implies a solution appropriate to the context: wearable, affordable in multiple units, functionally reliable, and cost-effective to analyze. Based on these criteria, physiological signals recorded were electrocardiography (ECG) and electrodermal activity (EDA). HRV features were derived from the ECG and used in the analysis. For work-type applications, cardiac metrics such as HRV features tend to be reactive on a time-scale of about a minute, and are useful for assessing activation and workload. Statistics of phasic skin conductance responses (SCRs) from EDA are complementary to HRV—they react on time-scale of seconds, are responsive mainly to external stimuli, and are commonly used to index emotional arousal. Unfortunately excessive data was lost due to device failure and thus EDA is not analyzed at a group level in the main paper. In the end of the paper, we discuss EDA and the requirements of field recordings as issues for future work. Physiological compliance was calculated as correlation between the same feature from each member of each pair. We also analyzed physiological compliance in relation to individual’s subjective ratings of the classroom session.

**Outline** In the rest of the paper, we first describe the related background literature. Then the Methods section sets out the design of the study including participants, protocol, and a our analysis approach. In the Results section we show that measuring physiological compliance in an uncontrolled naturalistic setting is feasible, for a robust sensor; interesting, because compliance is evident in collaborating dyads for a measure of mental workload but not for a measure of physical activity; relevant, because compliance predicts the subjective rating of demand in programming assignments. Finally we discuss the limitations of the results, and implications for applied and future work.

### Related work

**Pair-programming background** Collaboration in knowledge work in general is a well-studied topic the findings suggest that group productivity and collective mood converges over time [5]. The subjective quality of the collaboration is important. For example, in a self-report based study of N = 122 software projects from 29 companies, [6] found that intra-team social empathy predicted project success. Social empathy was moderated by the existence of group norms. Thus, the degree to which team members understand and accept each others’ point of view is important to collaborative function.

In our study collaboration is exemplified by pair-programming, the exercise of two programmers tackling on a task at a single computer [7]. Pair-programming is used both within the academia as a collaborative learning approach for novices learning to program [8], as well as within the industry to help produce higher quality code and to spread the tacit knowledge of experienced programmers inside organizations [9]. However, it is not established that pair-programming is always effective and useful. Pairing takes effort and has an adjustment period during which the productivity of the pair is lower than the productivity of the individuals [10]. Dyads can also be malformed, such that skill differentials, task-understanding, individual agreeableness, or some other factor will inhibit the productivity of one or both programmers [11, 12]. Indeed the number of possible factors which can affect a socially-interacting working partnership is so large that it may be better to look for measures of interaction success than to identify causes of failure.

Chaparro *et al* [13] studied the question *why is pair-programming sometimes ineffective?*, finding that matching by skill level and task demand are main contributing factors of success. Additionally, as in the team study cited [6], the efficacy of pair-programming also relies on good social interaction, as shown in [14]. The influence of subjective factors on collaboration implies the value of and requirement for developing objective measurement of social compliance, as enabled by physiological measurements. Such measurement could, for example, be used in-situ to assess the functionality of the pair and to provide feedback and improvement suggestions if needed.

**HRV and cognition background** HRV has been found to be reactive to mental workload and stress [15] in multiple contexts, e.g., various tests requiring executive functions [16], during aircraft piloting [17], and in computer work [18, 19]. Among others, Thayer *et al* [20] and Lane *et al* [21] have described integrative models of neural structure and autonomic regulation, and presented supporting studies which link cognition and HRV. These models suggest prefrontal cortical modulation on HRV parameters which is supported by the inhibitory role of prefrontal cortex via the vagus [22].

HRV research typically focuses on either long- or short-term recordings, of 1+ days or minutes/hours, respectively. Our protocol is short-term and so our methods derive from that literature. HRV analysis can use time-domain and frequency-domain metrics [23]. The time window recommended for short-term frequency-domain HRV metrics is 300 seconds [23] which makes it difficult to assess HRV with respect to short duration events, such as programming activity. On the other hand, frequency-domain metrics can reliably indicate the phenomenon of *sympathovagal balance*, that links HRV to cognition [23]. To use time-domain metrics for the same purpose, they must be carefully chosen [24].

Here we use time-domain metrics that have been shown to give reliable results in as short as 60 second time windows [25, 26]. All metrics are based on finding peaks of the ‘QRS’ complex wave that represents a heart beat in ECG, in the given time window. We calculate heart rate (HR) as the inverse of mean interbeat-interval (IBI, i.e., peak to peak delay in QRS complex), to use as an index of general activation. We use two other HRV metrics, rMSSD [24, 27], and especially SDNN [28], which have shown promise in expressing sympathovagal balance. SDNN is the standard deviation of IBI, where abnormal beats are not counted, thus giving the term NN for ‘normal-to-normal’. rMSSD is the mean square of successive differences between subsequent peaks.

**Physiological compliance background** It is well-known that social interaction tends to induce imitative behavior [29], even when the meaning of the behavior is unknown to the imitator [30, 31]. Imitative behavior has been proposed to fit within an ideomotor framework, where an intention to act precedes the action, as opposed to a pure stimulus-response model [32]. This in turn implies that a) imitative behavior is paralleled by imitative psychology, and b) such processes are reciprocal as each participant influences and is influenced by the other.

Gottman [33] provided an early overview and described methods to measure *cyclicity* in social interaction, from qualitative data. The approach was developed further in succeeding decades, and extended to include physiological signals. The term *social psychophysiological compliance* (SPC) was coined by Henning [4], and proposed as an index of collaborative performance [34]. Henning [4] suggests that due to their feed-forward influence of future behaviors, physiological changes during social interaction should be considered more than just a response to ongoing social behaviors. [4] proposes that physiological compliance benefits a social process in accordance with the social cybernetic model. This model asserts that SPC occurs before behavior [35, 36], in agreement with the ideomotor model. Thus, SPC refers to correlation between physiological measures of individuals, which arises over the course of interaction due to reciprocal changes in participants’ internal physiology [35, 37].

It has further been shown that SPC is dose-dependent on the intensity of social interaction. Leeuwen [38] showed that fetal HR synchronizes with mother’s respiration. Compliance of HRV and EDA were shown to react to intensity of social interaction in [39]. Compliance has found to be higher during competitive computer game play than cooperative play in a study by Chanel *et al*. [40], and the result has been replicated by Spape *et al*. [41]. Compliance is also found during a movie watching protocol [42]. Elkins *et al*. [37] examined methods of measuring SPC in HRV data as a predictor of team performance. They found that linear correlation and directional agreement were the most sensitive to performance, and greater intra-team SPC associated with better performance. This study is doubly relevant because the task itself was not stationary but complex in nature.

To summarize our literature review, field-measurements of collaboration quality are supported by convergent evidence from prior work on pair-programming, HRV and cognition, and physiological compliance.

## Methods

**Research Questions** Ordinary everyday work is done in noisy, uncontrolled settings, so our first research question RQ1 asks whether it is feasible to extract any SPC signal in such a setting (using our methods)? To address RQ1, we recorded ECG in a noisy naturalistic setting using wearable sensors. Based on correlations of ECG features, we contrast the correlations within dyads with other possible combinations of the recorded physiological signals recorded in the same class. This separates the influence of task related events from the stimuli irrelevant to the phenomena under study (e.g., experimenter instructions).

Secondly, RQ2, we ask whether the extracted SPC signal is relevant to some interesting task, or does the shared environment ‘drive’ the SPC to the exclusion of any detectable response to shared work? To address RQ2, we use the same analysis as for RQ1, but now contrast the HR correlation with correlation of SDNN and rMSSD features. This contrasts the relative compliance of physical activation with specific cognitive activation or mental workload.

Collaboration is an essentially inter-subjective phenomenon [43]. Thus we pose RQ3 as a supplement to RQ2: do participants themselves agree with the SPC signal? To address RQ3 we ask the participants to individually report their task demands using the NASA Task Load Index [44], and construct a regression model to predict the SPC (using HRV features).

Finally, it is also desirable to investigate the causes or task-based correlates of the observed SPC, and thus say what is it useful for. However to remain naturalistic, the assignments were taken from the course curriculum without manipulation. Thus participants’ development on the assignments was uncontrolled and we lacked robust classifiers to study such relations.

- H1.feas—correlation of HRV features will be greater for signals of collaborating dyads, giving measurable SPC.
- H2.intr—measurable SPC will relate to activity of interest by the contrast of task-relevance of ECG features HR (physical activation) versus SDNN or rMSSD (mental activation).
- H3.rele—participants’ self-assessment of task-relevant activity will predict the SPC *of interest*, thus HRV features will predict items from NASA TLX.

### Study Design

Our design takes place in a class room environment for novice student programmers, overseen by experimenters using a single laptop for timing and notes. The study protocol has been approved by the ethical review board of Hospital District of Helsinki and Uusimaa, Finland. Study procedures followed the guidelines of the Declaration of Helsinki for human experiments.

All students are first briefed together and give their written informed consent. Sensors are then attached—every participant is fitted with a wearable medical grade device for recording ECG (eMotion Faros 180°, Mega Electronics Ltd). The ECG electrode placements are on right coracoid process and on the lower left rib-cage. The Faros device clocks are pre-synchronized with the experimenter’s laptop that controls the timing of the measurement protocol. Physiological measurements start immediately after attachment, and end only when sensors are detached.

Participant dyads are seated together in order of arrival, then all students first watch a baseline video together. Each dyad proceeds to work on standard assignments from their curriculum, on one workstation, where dyad members swap roles between ‘driving’, i.e., active programming, and ‘navigating’, i.e., guiding and commenting, in a typical pair-programming design. The task requires cooperation between dyad members to accomplish a shared goal.

Each dyad sets their own pace for processing the assignment. This implies that the degree of cooperation is left to participants, allowing for a variety of SPC and performance scores. Whereas if the pace is forced by the protocol, this ‘artificial influence’ would be likely to account for some of the observed SPC. Dyads swap the role of driver and navigator every 7 minutes, the baseline video lasts 7 minutes, and with preparation total experiment time is about 90 minutes. Timestamps for all such activity is recorded on the central experimenters’ laptop.

**Fig 1:**
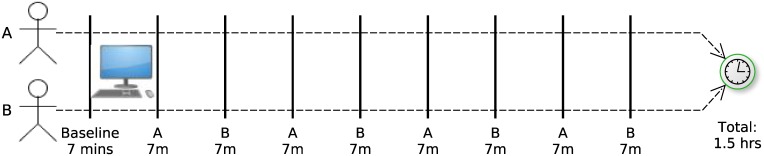
The pair-programming protocol. 7min baseline video. Segments labeled A, 7mins: participant A drives, participant B navigates. Segments labeled B, 7mins: participant B drives, participant A navigates.

The task in the class consists of two separate assignments. The programming assignments are counter balanced within each class, so half the dyads started with one and half with the other assignment. Assignments are completed using the NetBeans for Java integrated development environment (IDE, https://netbeans.org/). The experiment protocol for one dyad is illustrated in Fig 1.

### Participants and Recordings

46 student participants were recruited by advertisement through class mailing list, and remunerated with movie tickets. After examining the ECG data for artefacts data of 38 participants remained for subsequent analysis. In this set of 38, there were 16 females, 22 males, three left-handed, with age range: 28 from 18–23 years, 9 from 24–29 and one was over 34.

Recording sessions were conducted by authors 1 and 3 with assistance from the course instructor, author 5. The class participated is part of the programming course and differed from ordinary programming session only by the physiological measurements. The participants were told not to pay attention to the measurement devices or experimenters. After the experiment, participants were asked to report on their state: hours slept M = 7.6 (SD = 1.64), hours awake M = 4.8 (SD = 1.53), current sleepiness (by KSS Karolinska Sleepiness Scale, 9-points 1 = alert 9 = sleepy) M = 4.5 (SD = 2.19). They were also asked to rate the exercise according to the NASA Task Load Index (TLX) [44], a validated instrument with 11 point scales for various aspects of task effort (physical demand is excluded from reporting as irrelevant):

- ‘mental demand’ (MD) M = 6.0 (SD = 2.05) (0: not demanding, 10: demanding)
- ‘temporal demand’ (TD) M = 6.0 (SD = 1.88) (0: not demanding, 10: demanding)
- ‘frustration’ (Fr) M = 4.2 (SD = 2.91) (0: not frustrated, 10: frustrated)
- ‘performance’ (Pe) M = 6.4 (SD = 2.50) (0: performed badly, 10: performed well)
- ‘effort’ (Ef) M = 6.3 (SD = 2.62) (0: did not need effort, 10: needed effort)

Finally an extra item was asked alongside TLX to assess the concentration while navigating (partner was driving), on an 11-point scale:

- ‘concentration while navigating’ (Na) M = 8.1 (SD = 1.69) (0: no collaboration, 10: intensive collaboration)

For every collaborating dyad we also compute the sum of all self-report items. These are shown in Table 1 together with information whether the pair was all-male (MM), all-female (FF), or mixed (MF).

**Table 1:** Sum of TLX questionnaire results in each dyad, with corresponding HR, SDNN and rMSSD correlation in 60 and 300 second windows. Sorted in decreasing order of SDNN in 300 second window. MD is mental demand, TD temporal demand, Pe performance, Ef effort, Fr frustration, and Na concentration while navigating.

							60			300		
Sex	MD	TH	Pe	Ef	Fr	Na	HR	SDNN	rMSSD	HR	SDNN	rMSSD
MM	10	11	16	15	6	17	0.56	0.18	0.22	0.77	0.65	0.64
FF	10	12	10	14	11	13	0.19	0.38	-0.04	0.18	0.64	-0.02
MM	9	8	15	8	6	16	0.14	0.11	0.26	0.03	0.58	0.71
MF	11	11	12	15	14	17	0.63	0.38	0.18	0.80	0.56	0.23
MF	16	12	9	17	15	17	0.30	0.09	0.12	0.61	0.53	-0.22
MM	16	11	14	11	3	17	0.47	0.14	0.18	0.73	0.43	0.47
FF	13	11	9	15	17	16	-0.13	0.18	-0.05	-0.26	0.42	-0.24
MM	15	9	18	10	2	17	0.47	0.25	0.28	0.67	0.42	0.77
MF	10	10	9	10	7	15	0.32	0.04	0.14	0.37	0.41	0.06
FF	8	16	19	6	2	17	0.12	0.25	-0.18	-0.07	0.33	-0.54
MF	16	14	14	14	10	19	0.25	0.37	0.08	-0.09	0.33	0.18
MM	7	13	13	4	1	18	0.35	0.03	0.29	0.43	0.24	0.50
MF	14	11	10	16	11	12	0.01	0.28	0.17	-0.34	0.19	0.40
MF	12	11	15	17	10	20	0.11	0.22	0.15	0.02	0.15	0.50
MF	13	13	14	13	11	17	-0.20	0.09	-0.28	-0.42	0.15	-0.35
MM	13	14	18	11	4	19	-0.13	-0.01	-0.06	-0.60	0.00	-0.37
MF	11	11	9	10	9	10	0.37	0.16	0.31	0.67	-0.02	0.63
MF	13	14	12	15	8	17	0.34	0.13	-0.17	0.38	-0.11	-0.33
MF	12	16	6	18	14	15	0.31	-0.17	-0.16	0.35	-0.39	-0.27

### SPC calculation and testing

The ECG signal was preprocessed in R [45] using Colibri R-package [46]. The R-peaks were automatically detected from ECG and used to form the IBI series that were used as a basis for HRV analyses. The calculation of HRV features was performed in sliding time windows with a length ranging from 60 to 300 seconds, using an overlap of 1/3 of the window length. The window lengths were chosen according to Smith *et al*, who suggested that shortest reliable window for time-domain HRV features is 60 seconds [27].

Features extracted were HR, SDNN, and rMSSD. These were computed according to their standard definitions. For a given window length, we obtain three feature vectors for every participant, denoted $x_{i}^{HR}$, $x_{i}^{SDNN}$, and $x_{i}^{rMSSD}$, where *i* ∈ {1, 2, …, 38} is a participant identifier. We will omit the feature name from the superscript unless these are unclear from the context. For every feature, *x*_*i*_(*t*) is the value of the feature in the *t*:th time window.

We used the permutation testing framework to test H1.feas and H2.intr, see, e.g., [47] for details and discussion. As test statistics we used Pearson’s product-moment correlation coefficients of the feature vectors within the dyads, i.e., pairs of participants that collaborated in the classroom, with randomly generated pairs of physiological signals, as follows. Every dyad consists of two participants, denoted by identifiers (*i*, *j*), where *i* ranges from 1 to 19, and *j* = *i* + 19. I.e., the dyads are the pairs (1, 20), (2, 21), …(19, 38). We compute the values $cor(x_{i}^{F},x_{j}^{F})$ for every dyad, where *F* is either HR, SDNN, or rMSSD, and *cor*(⋅, ⋅) is Pearsons’ correlation coefficient. The average correlation of the dyads, denoted *μ*_*true*_, is the arithmetic mean of these pairwise correlations. We use *μ*_*true*_ as an estimate of SPC for collaborating members of pairs in the subsequent analysis. The pair-specific values of HR, SDNN, and rMSSD for window lengths of 60 and 300 seconds are shown in Table 1.

Our null hypothesis was that the average correlations of dyads, as defined above, would not be affected if the pairs would be randomly shuffled within the class room and the correlations would be computed for the shuffled pairs. The pairs of signals are formed by permuting the second signal across all possible signals measured. More formally, the set of signals are the pairs (1, *r*_1_), (2, *r*_2_), …, (19, *r*_19_), where the values *r*_1_, *r*_2_, …, *r*_19_ form a permutation of the integers 20, 21, …, 38, with restrictions for second signal to be in the same class. The permutation *r* is drawn *uniformly at random*. Given a permutation *r*, we compute the values *cor*(*x*_*i*_, *x*_*r*_*i*__) for every shuffled pair, and use their arithmetic mean, denoted *μ*_*r*_, as a sample from the null hypothesis.

The pairs in the null distribution thus all contain one driving and one navigating partner from the same room. Any evidence of SPC that we observe for the the pairs sampled from null hypothesis should be caused by confounders in the environment instead of collaboration. The random set of correlations was computed 10000 times to generate the distribution of *μ*_*r*_. This is done by generating a different permutation *r* 10000 times. The obtained distribution sets confidence intervals (CIs) for the *μ*_*r*_ and allows us to compute our two-tailed p-values for the true correlation averages (*μ*_*true*_) [47]. This process is conducted in the same manner for all three features (HR, SDNN, rMSSD) and several window lengths (from 60 to 300, in 40 second steps).

Finally, H3.rele was tested by fitting a linear regression model to study the association between SPC and the self-report items in the dyads. This was done for all three features (HR, SDNN, rMSSD) and two window lengths (60 and 300). Predictors in each model are pairwise sums in the dyads of the self-report items (shown in Table 1). SPC, i.e., the Pearson correlation, is the dependent variable. We have thus

$$\begin{array}{c} cor\left(x_{i}^{F},x_{j}^{F}\right)∼\beta_{0}+\beta_{m}{\mathrm{MD}}_{ij}+\beta_{t}{\mathrm{TD}}_{ij}+\beta_{p}{\mathrm{Pe}}_{ij}+\beta_{e}{\mathrm{Ef}}_{ij}+\beta_{f}{\mathrm{Fr}}_{ij}+\beta_{n}{\mathrm{Na}}_{ij} \end{array}$$ (1)

over all dyads (*i*, *j*), with *F* being one of HR, SDNN and rMSSD, and *X*_*ij*_ denotes the sum of the corresponding self-report item *X* for participants *i* and *j* and *β*_*x*_ its coefficient.

## Results

We found support for H1.feas from the result that the SDNN correlation for dyads is significantly greater than that for the null hypothesis. The result is robust across all tested window lengths (the data and examples can be found in S1, S2 and S3 Files). Table 2 shows the position of mean dyad correlations with respect to the 95% confidence intervals (CIs) in the null hypothesis. For SDNN the highest correlations and greatest difference from the null hypothesis occur in 300 second window length with a Holm-Bonferroni corrected *p*-value of 0.007.

**Table 2:** For HR, SDNN, rMSSD across window lengths from 60 to 300 seconds in 40 second increments: 95% CIs for shuffled pair correlation distribution, means for the collaborating dyad correlation, and tests of significance.

		shuffled pairs		dyad	
Window length	ECG feature	2.5%	97.5%	Mean cor	*p* -value adj.
**60 sec**	HR	0.108	0.308	0.235	0.309
	rMSSD	-0.061	0.083	0.076	0.081
	SDNN	0.017	0.146	0.162	0.022 *
**100 sec**	HR	0.118	0.357	0.273	0.302
	rMSSD	-0.088	0.099	0.078	0.121
	SDNN	0.005	0.17	0.181	0.049 *
**140 sec**	HR	0.113	0.378	0.253	0.503
	rMSSD	-0.103	0.134	0.108	0.125
	SDNN	0.008	0.209	0.243	0.009 **
**180 sec**	HR	0.085	0.392	0.24	0.530
	rMSSD	-0.111	0.134	0.103	0.140
	SDNN	0.008	0.226	0.246	0.031 *
**220 sec**	HR	0.054	0.389	0.222	0.529
	rMSSD	-0.13	0.146	0.125	0.089
	SDNN	0.002	0.23	0.239	0.051 •
**260 sec**	HR	0.083	0.427	0.251	0.531
	rMSSD	-0.11	0.2	0.156	0.155
	SDNN	-0.034	0.219	0.269	0.007 **
**300 sec**	HR	0.046	0.398	0.221	0.522
	rMSSD	-0.141	0.19	0.145	0.157
	SDNN	-0.04	0.232	0.289	0.007 **

Holm-Bonferroni corrected *p*-values test whether the collaborating dyad correlation average is from average correlation distribution for the shuffled pairs; significance levels indicated as *p*<0.1 •, *p*<0.05 *, *p*<0.01 **

Table 2 also illustrates support for H2.intr. As compared to SDNN correlations, all mean dyad HR correlations are well inside the 95% CIs for the null hypothesis. This shows that in each recording session, the correlation of HR-indexed physical activation was insensitive to the pairing of individuals, contrasting with SDNN-indexed cognitive activation. Therefore the *only* feasible measure of SPC comes from SDNN, a HRV feature related to cognition, and not from physical activation.

Support for H3.rele is clear from the contrast between models for HR versus SDNN and rMSSD features, as shown in Table 3. The mean HR did not express linear dependencies on self reports. Self-reported performance explains some of the variance in SDNN at 60 second window, however, the adjusted *R*^2^ is small. In the 300 second window the model explains some variation for SDNN (*R*^2^ = 0.20) and is significant for self-reported temporal demand. The relationship of rMSSD to self-reported temporal demand (check the Table 1) was highly significant and explains a large fraction of the variation (*R*^2^ = 0.42 for the 60 second time window and *R*^2^ = 0.50 for the 300 second window).

**Table 3:** Regression model of computed HRV features and self-reports. Collaborating dyad correlations were computed for 60 and 300 seconds, and fitted by self-report scores summed for each dyad. Column headers, from left: **Win.** window length of analysis; HRV **Feature**; **adj.** *R*^2^ adjusted R-squared value for the linear model; **Fitted** linear model parameter, i.e., the questionnaire item; **Est.** model estimate; **Std.Err.** model standard error; and confidence intervals for model predicted linear dependency.

Win.	Feature	adj. *R*^2^	Fitted	Est.	Std.Err.	2.5% − 97.5%	
60	HR	-0.09	(Intercept)	0.96	0.56	-0.26	2.17
			Mental	-0.01	0.03	-0.07	0.04
			Performance	-0.04	0.03	-0.10	0.02
			Effort	0.03	0.03	-0.03	0.10
			Temporal	-0.03	0.03	-0.09	0.03
			Frustration	-0.05	0.03	-0.10	0.01
			Navigating	0.02	0.03	-0.05	0.09
300	HR	-0.07	(Intercept)	1.93	1.04	-0.34	4.20
			Mental	-0.02	0.05	-0.13	0.09
			Performance	-0.08	0.05	-0.20	0.03
			Effort	0.05	0.05	-0.07	0.17
			Temporal	-0.07	0.05	-0.18	0.05
			Frustration	-0.08	0.05	-0.18	0.02
			Navigating	0.03	0.06	-0.10	0.16
60	SDNN	0.04	(Intercept)	0.03	0.32	-0.66	0.72
			Mental	0.00	0.01	-0.03	0.04
			Performance	0.04	0.02	0.00	0.07*
			Effort	-0.00	0.02	-0.04	0.03
			Temporal	-0.01	0.02	-0.05	0.02
			Frustration	0.02	0.01	-0.01	0.05
			Navigating	-0.02	0.02	-0.06	0.02
300	SDNN	0.20	(Intercept)	0.77	0.57	-0.48	2.02
			Mental	-0.01	0.03	-0.07	0.05
			Performance	0.03	0.03	-0.03	0.09
			Effort	-0.02	0.03	-0.08	0.05
			Temporal	-0.08	0.03	-0.14	-0.01*
			Frustration	0.03	0.03	-0.03	0.09
			Navigating	0.01	0.03	-0.06	0.08
60	rMSSD	0.42	(Intercept)	1.09	0.32	0.39	1.78
			Mental	0.00	0.02	-0.03	0.03
			Performance	-0.02	0.02	-0.05	0.02
			Effort	0.00	0.02	-0.03	0.04
			Temporal	-0.06	0.02	-0.10	-0.03**
			Frustration	-0.02	0.01	-0.05	0.01
			Navigating	0.01	0.02	-0.03	0.05
300	rMSSD	0.50	(Intercept)	2.48	0.70	0.96	3.99
			Mental	-0.01	0.03	-0.09	0.06
			Performance	-0.02	0.04	-0.10	0.06
			Effort	0.04	0.04	-0.04	0.12
			Temporal	-0.15	0.03	-0.22	-0.07**
			Frustration	-0.06	0.03	-0.13	0.01
			Navigating	-0.01	0.04	-0.09	0.08

Adjusted model CIs that do not include 0 denote a linear dependency is found.

Confidence levels shown as * *p*<0.05, ** *p*<0.01.

## Discussion

We have demonstrated support for three hypotheses regarding the feasibility, interest and relevance of recording an SPC signal in a noisy, naturalistic setting.

The SDNN correlation shows significant difference in collaborating participants compared to the average over the classroom, across all window lengths from canonical 300 to minimum 60 seconds (only marginal, however, for 220 seconds window). This contrasts with the HR parameter which is high for all pairs regardless if the participants worked together or not. Also for rMSSD there is some effect in correlations for collaborating participants. The effect is smaller than for SDNN, and does not test as significant, but the collaborating dyad average correlation lies in the highest quartile of the shuffled pair correlation distribution across all windows. This could be relevant to expanding these results to the time-domain correspondent of sympathovagal balance [25] in further work.

Our use of time domain alternatives to the classical frequency space HRV parameters allow us to calculate the feature with a shorter time window. We validate that the compliance findings, i.e., feasible, interesting, and relevant correlations, apply across different window lengths. Our results thus develop the study of collaboration in ecologically valid setups with no fixed-time design (as is usual in ECG-paradigms).

**Fig 2:**
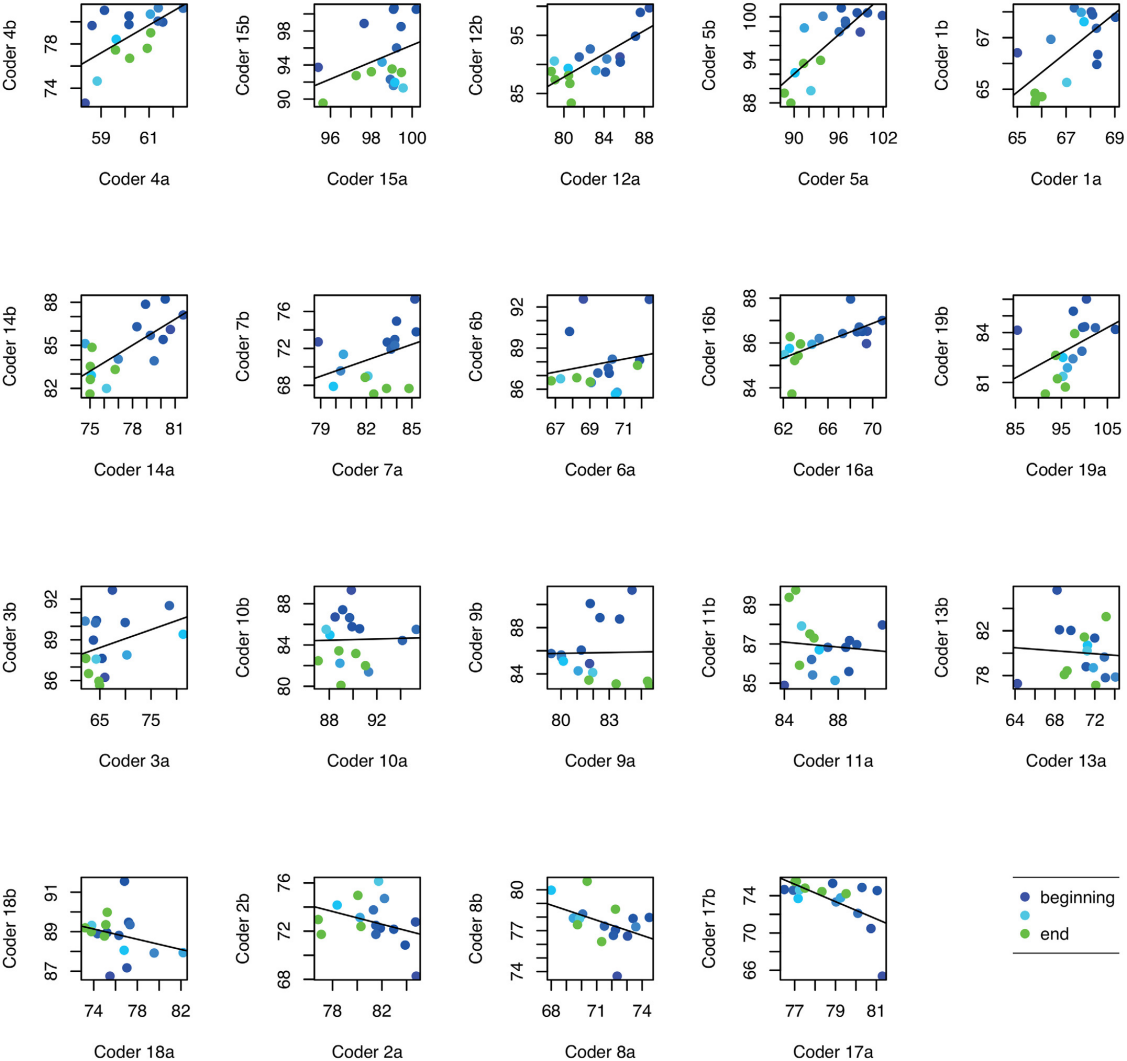
Pairwise correlations for mean HR in 300 second windows over the session. Line in each subplot depicts the regression between each dyad. Color changes blue to green from the beginning to the end of the session.

**Fig 3:**
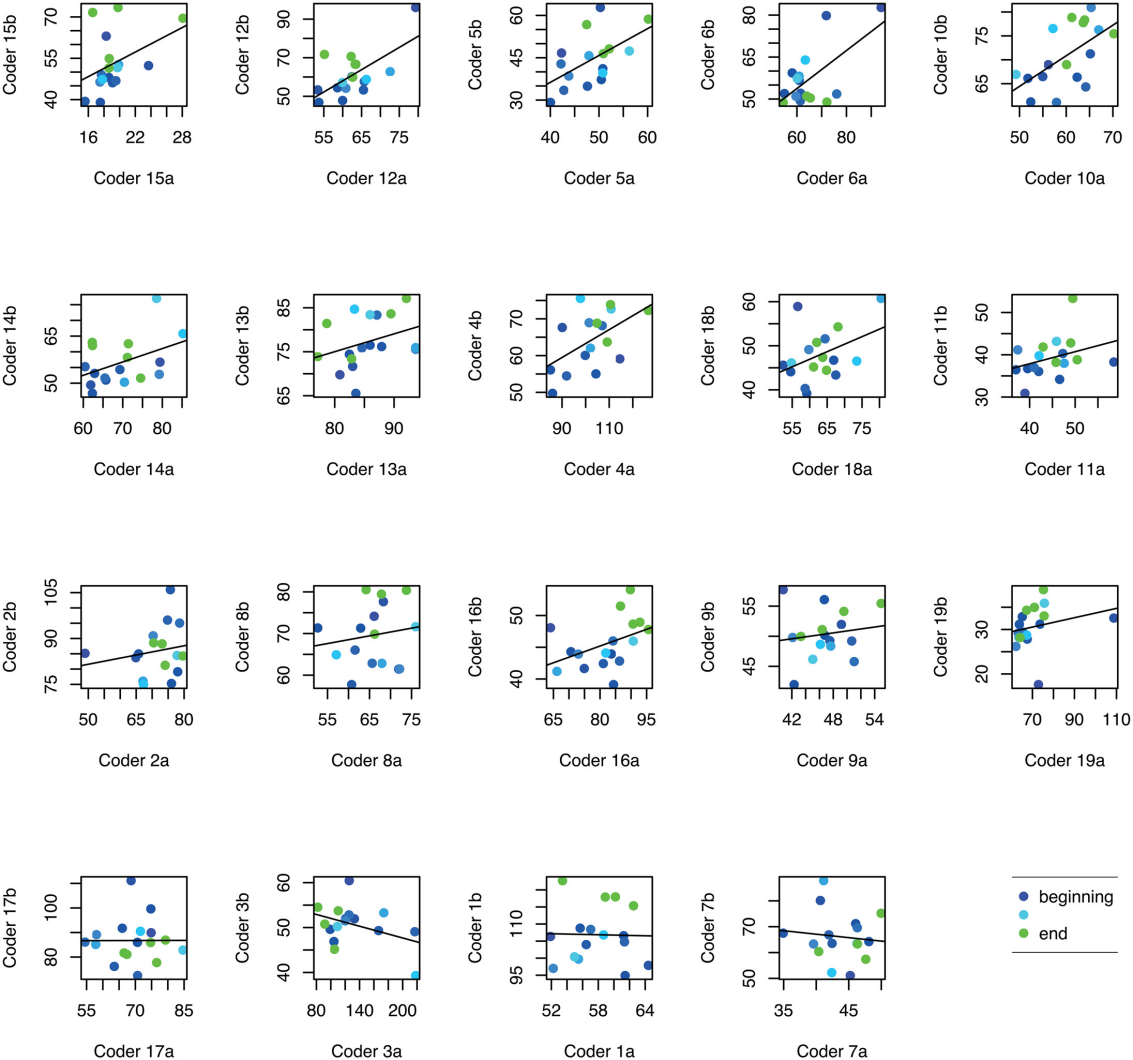
Pairwise correlations for SDNN feature in 300 second windows over the session. Line in each subplot depicts the regression between each dyad. Color changes blue to green from the beginning to the end of the session.

**Interpretations** The high correlations in HR, over all dyads and shuffled pairs, could be explained by a decreasing trend in HR data during the session for 79% of the dyads. In contrast, for SDNN less than half of the participants expressed negative linear trend during the measurement session. Figs 2 and 3 show HR and SDNN correlations for all 300 second time windows during the session, separately for each dyad. Each point represents the advancing time windows across the practice session using a color spectrum from blue in the beginning to green at the end. Subplots are sorted by the linear regression fit lines (although note that axis scaling varies).

The contrast of time-dependent physical activation with time-independent cognitive activation suggests distinct sources of influence. In other words, pairwise HR correlations may be (at least partly) driven by a generally shared linear physiological process, e.g., becoming more physically relaxed over time. The elevated SDNN synchrony within the collaborating participants is more likely to reflect dyad-specific processes, i.e., each dyad’s own task-related progress and behavior.

SPC for SDNN and rMSSD depended linearly on self-reported temporal demand, with negative relation, i.e., less demand is felt when SPC increased. With SDNN this dependence appears only in longer analysis window lengths. The mild positive relation with performance and SDNN correlation was only observed in short 60 second time-window, which in general provided less stable results.

**Limitations and future work** The results demonstrate the efficacy of ECG and the relevance of other HRV parameters over HR for SPC. However, the current analysis cannot account for *why* the SPC was observed.

SPC did relate to self-reported performance and temporal demand. Thus the results are at least influenced by relevant subjective processes. Nevertheless, tasks presumably also contain features that influence SPC but are unrelated to relevant processes. Such latent variables could include, for example, the dyads’ history of collaboration, because we did not control for self-selection of pre-acquainted dyads by the participants. To address the issue properly, future work needs more rigorous control or at least observation of task-dyad interaction features, such as history and relative programming skill per participant. Also, the SPC relations to self-reported items should be further studied by including more check-points during future recordings.

To investigate productivity, we attempted to analyze assignment completion rates with respect to all HRV features. However analysis was exploratory, as we had no hypothesis for task-physiology relationship in a naturalistic setting with a novel design. Analysis gave no results of interest, perhaps because only half of dyads completed at least one assignment which resulted in a lack of variance in the data. This could be resolved if, in future work, assignments are broken into smaller components which can be individually assessed.

High-resolution behavioral data can be obtained with keyloggers, for example the ‘Test My Code’ (TMC) plugin [48] for the NetBeans IDE. The TMC plugin records the timestamp and change for each key-press within the programming environment, and automatically links the information to the programming task at hand. However, the raw signal from this behavioural data is rapidly changing, and as noted the time resolution of HRV parameters is insufficient to study such rapid changes. To relate physiology to a task like pair-programming requires a better match between resolutions of each signal, and better contingency of the physiological signal.

EDA is a good candidate signal for phasic analysis of programming, because the phasic component has time resolution of ∼1 second, and is contingent to external stimuli. Loss of data prevented such analysis on a group level in this study. However the sparse data retrieved already justify further work, as discussed in S1 Appendix. This supplement is included, not to claim additional results, but instead show the potential of the EDA approach to address the issues not addressed in the reported experiment. The potential is clear from the few successful EDA measurements illustrated in S1 Fig. It therefore serves as both a motivation and a guideline for future work.

The data lost in behavioral and physiological modes (eight ECG sets and almost all EDA) illustrates the fragility of naturalistic paradigm to recording issues, motivating more efforts to create robust integrated field-ready setups. Thus future work will focus on these problems and establishing a link between SPC and task performance. If greater SPC is found to be associated with better performance, the result could lead to applications for improved team training and assessment.

**Conclusion** The current paradigm suggests that by measuring HRV with simple ambulatory ECG device in an ecologically valid pair-programming setting, physiological signals can provide information on SPC. Specifically, it is reasonable to extract the SPC signal; given such a signal, we can differentiate between environmental influence and task-relevant influence. The SPC resulting from collaboration can be detected from the physiological signals and the signals also depend on self-reported demands of the session. Future work will add insight into the relationship between physiology and behavior by also recording EDA and keylogger data, and adding granularity in assignment structure, to increase the number of data points in time.

## Supporting Information

S1 Appendix Report on EDA data recorded in the measurements. Information on four (two dyads) successful EDA recordings in the paradigm. Appendix exemplifies the potential in using EDA signals for compliance assessment in field studies.
(PDF)

S1 Fig EDA results from example dyads. (TIF)

S1 File The ECG signals part 1. (ZIP)

S2 File The ECG signals part 2. (ZIP)

S3 File Example script and questionnaire data. Scripts for replicating the results.
(ZIP)
