# Supplementary material for: Cognitive Collaboration Found in Cardiac Physiology: Study in Classroom Environment
Source: PLoS One. 2016 Jul 14;11(7):e0159178. doi: 10.1371/journal.pone.0159178 (PMC4944990; doi:10.1371/journal.pone.0159178)
Supplement: S1 Appendix — Information on four (two dyads) successful EDA recordings in the paradigm. Appendix exemplifies the potential in using EDA signals for compliance assessment in field studies. (PDF) [file pone.0159178.s001.pdf]

S1: Analysis of Electrodermal Activity Recordings in Pair Programming from 2 Dyads

Lauri Ahonen[1,*], Benjamin Cowley[1,3], Jari Torniainen[1], Antti Ukkonen[1], Arto Vihavainen[2], Kai Puolamäki[1] [1]

[1]Finnish Institute of Occupational Health, Finland

[2]Department of Computer Science, University of Helsinki, Finland

[3]Cognitive Brain Research Unit, Institute of Behavioural Sciences, University of Helsinki, Finland

July 1, 2016

1 Introduction

As mentioned in Introduction, electrodermal activity (EDA) was also recorded during the pair programming sessions. EDA is a valuable addition to almost any psychophysiological experiment due to its ability to measure sympathetic arousal without being influenced by parasympathetic activation. Contrasted to the heart-rate variability (HRV) which is controlled by both branches of the autonomic nervous system (ANS), the combination of EDA and HRV produces the most complete picture of the ANS responses. EDA is temporally more responsive when compared to HRV as responses typically occur within 1-3 s after a stimulus onset [3] where as the time-scale for HRV is in minute-scale.

Unfortunately a large portion of the collected EDA data had to be discarded due to excessive artifacts in the data. However, we report here visual analysis

of two of the successfully collected dyads.

2 Methods, Results, and Discussion

The EDA was recorded in the experiment using the E4 wearable sensor (Empatica Inc., MA, USA) [4] attached to the wrist of the non-dominant hand. The stainless steel electrodes were housed in the wrist-strap of the device and positioned on the ventral side of the wrist. The signal quality was first confirmed visually by streaming the data over bluetooth but during the session the data was stored in the on-board memory of the device for offline analysis. The signal was registered at 4 Hz and synchronized with other measurement devices.

The collected EDA data was subjected to standard analysis by first detecting and correcting motion induced artifacts through interpolation and then decomposed into phasic and tonic components through Continuous Decomposition Analysis (CDA) [1]. The phasic and tonic components represent the skin conductance response (SCR) and skin conductance level (SCL) portions of the signal respectively. Both artifact correction and CDA were performed in MATLAB (MathWorks, Natick, MA, USA) using the Ledalab-toolbox (`http://ledalab.de`) Finally the significant peaks of the phasic response were compared to the time course of the session. The detection threshold for significant peaks was set to $0.25\mu S$ as suggested by the E4 manual.

Phasic component represents the arousal reaction to external stimuli and emotionally arousing conditions whereas the tonic component describes the overall state of sympathetic arousal. Fig. 1 displays the raw data, phasic component and the number of peaks from two dyads throughout the programming session.

From S1 Fig. 1 several observations can be made. First, all participants show some level of phasic activity throughout the session (bottom left panel) indicating that the setup itself was emotionally arousing. Second, the participant 01b is showing a clear pattern of increased phasic activity in coding blocks and reduced activity in navigation blocks (bottom left panel). This pattern is clearly seen

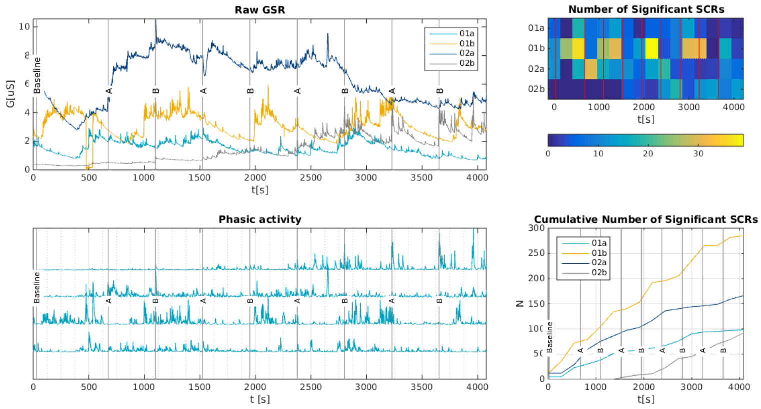

Figure 1: The abscissa of each panel shows time in seconds for the experiment. Vertical lines indicate phases of coding activity for each member of the dyad, A and B. **Top left:** raw EDA data in μS units for each member of the two dyads. **Bottom left:** phasic activity, or skin conductance responses (SCRs), the high frequency autonomic responding of the participants to external stimuli with the slow frequency tonic conductance level removed (μS units separated by participants). **Top right:** number of SCRs over threshold in a given block time, per participant. **Bottom right:** cumulative SCRs per participant.

in the number of significant SCRs for 01b (top right panel), which are greater than literature values for non-stimulating conditions (1-3 peaks/min) [2] in 'B' (driving) but not 'A' (navigating) blocks. Third, dyad 2 (participant 02a, 02b) showed a reciprocal pattern of activations throughout the session as 02a decreases and 02b increases, this could be attributed to the task. The observed results can be summarized as follows 1) task modulated activity can be seen in the EDA signal, 2) colocated cooperating participants nevertheless respond differently (in terms of pattern and not just degree) to task stimulus. Together with the increased temporal resolution, we believe that EDA could indeed be used to complement HRV when studying the physiology of pair programming.

References

[1] M. Benedek and C. Kaernbach. A continuous measure of phasic electrodermal activity. *Journal of neuroscience methods*, 190(1):80–91, 2010.

[2] W. Boucsein. *Electrodermal activity.* Springer Science & Business Media, 2012.

[3] M. E. Dawson, A. M. Schell, and D. L. Filion. 7 the electrodermal system. *Handbook of psychophysiology*, 159, 2007.

[4] M. Garbarino, M. Lai, D. Bender, R. W. Picard, and S. Tognetti. Empatica e3—a wearable wireless multi-sensor device for real-time computerized biofeedback and data acquisition. In *Wireless Mobile Communication and Healthcare (Mobihealth), 2014 EAI 4th International Conference on*, pages 39–42. IEEE, 2014.
