# Supplementary figures and images for: Cognitive Collaboration Found in Cardiac Physiology: Study in Classroom Environment
Source: PLoS One. 2016 Jul 14;11(7):e0159178. doi: 10.1371/journal.pone.0159178 (PMC4944990; doi:10.1371/journal.pone.0159178)

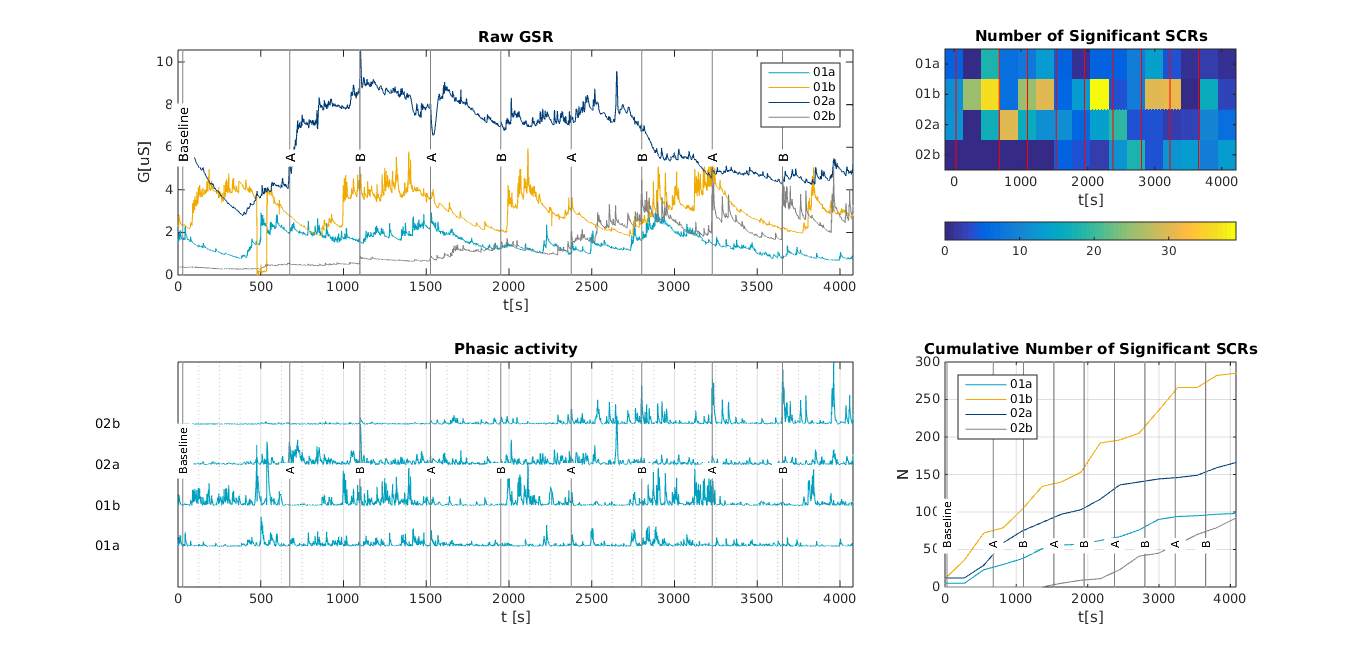

Supplement: S1 Fig — (TIF) [file pone.0159178.s002.tif]
